# A Prototype of a Neural, Powered, Transtibial Prosthesis for the Cat: Benchtop Characterization

**DOI:** 10.3389/fnins.2018.00471

**Published:** 2018-07-13

**Authors:** Hangue Park, Muhammad S. Islam, Martha A. Grover, Alexander N. Klishko, Boris I. Prilutsky, Stephen P. DeWeerth

**Affiliations:** ^1^School of Electrical and Computer Engineering, Georgia Institute of Technology, Atlanta, GA, United States; ^2^Biomechanics and Motor Control Laboratory, School of Biological Sciences, Georgia Institute of Technology, Atlanta, GA, United States; ^3^Wallace H. Coulter Department of Biomedical Engineering, Georgia Institute of Technology, Atlanta, GA, United States; ^4^School of Chemical & Biomolecular Engineering, Georgia Institute of Technology, Atlanta, GA, United States; ^5^P.C. Rossin College of Engineering and Applied Science, Lehigh University, Bethlehem, PA, United States

**Keywords:** bone-anchored transtibial prosthesis, sensing and powered prosthesis, closed-loop control, cat, ground reaction force

## Abstract

We developed a prototype of a neural, powered, transtibial prosthesis for the use in a feline model of prosthetic gait. The prosthesis was designed for attachment to a percutaneous porous titanium implant integrated with bone, skin, and residual nerves and muscles. In the benchtop testing, the prosthesis was fixed in a testing rig and subjected to rhythmic vertical displacements and interactions with the ground at a cadence corresponding to cat walking. Several prosthesis functions were evaluated. They included sensing ground contact, control of transitions between the finite states of prosthesis loading, and a closed-loop modulation of the linear actuator gain in each loading cycle. The prosthetic design parameters (prosthesis length = 55 mm, mass = 63 g, peak extension moment = 1 Nm) corresponded closely to those of the cat foot-ankle with distal shank and the peak ankle extension moment during level walking. The linear actuator operated the prosthetic ankle joint using inputs emulating myoelectric activity of residual muscles. The linear actuator gain was modulated in each cycle to minimize the difference between the peak of ground reaction forces (GRF) recorded by a ground force sensor and a target force value. The benchtop test results demonstrated a close agreement between the GRF peaks and patterns produced by the prosthesis and by cats during level walking.

## Introduction

Individuals with lower limb loss wearing a unilateral passive prosthesis frequently show asymmetric walking, which can lead to undesirable compensations and subsequent degenerative musculoskeletal conditions (Burke et al., 1978; Jaegers et al., 1995; Struyf et al., 2009). Among the variety of underlying reasons causing locomotor asymmetry, the inappropriate motor output and the lack of somatosensory feedback from the prosthetic limb are probably most important (Hof et al., 2007; Kannape and Herr, 2014). To correct these motor and sensory deficits, it is necessary to establish a bidirectional communication interface between the nervous system and the prosthesis.

Recent studies have shown the feasibility of replicating tactile sensory feedback from the amputated, phantom limb by electrical stimulation to residual cutaneous nerves (Dhillon et al., 2004; Ortiz-Catalan et al., 2014; Tan et al., 2014; Davis et al., 2016; Graczyk et al., 2016). Myoelectric signals with built-in pattern recognition algorithms enable fine motor control in arm prostheses, even without any sensory feedback (Li et al., 2010; Tkach et al., 2014). Likewise, it might be possible to improve locomotor outcome measures (e.g., walking symmetry) by controlling a powered prosthesis or orthosis using myoelectric signals from residual or intact muscles (Sawicki and Ferris, 2009; Herr and Grabowski, 2012; Takahashi et al., 2015; Kannape and Herr, 2016).

Recent developments of bone-anchored lower limb prostheses have improved the load transmission to the skeletal system, range of motion, comfort, and osseoperception (Hagberg and Branemark, 2009; Juhnke et al., 2015; Leijendekkers et al., 2016). In addition, bone-anchored limb prostheses may potentially allow for a secure and stable neural interface between the residual nerves and muscles and the prosthesis (Pitkin et al., 2012; Al-Ajam et al., 2013; Ortiz-Catalan et al., 2014).

We have used rodent and feline animal models to test integration of skin-and-bone integrated pylons (SBIP) with the residual tissue (Pitkin et al., 2009; Farrell et al., 2014b,c; Jarrell et al., 2018). These studies have demonstrated the potential of the SBIP implant to provide secure, infection-free fixation of the prosthesis to the residual limb. This type of implant can also be used as a gateway for transmission of nerve and myoelectric signals between the residual limb and prosthesis (Pitkin et al., 2012). For example, pressure applied to the prosthesis during the stance phase of walking can be transmitted to the nervous system via electrical stimulation of residual cutaneous nerves (Park et al., 2015, 2016), whereas myoelectric activity recorded in residual muscles can be used to drive prosthetic actuators.

Although bone-anchored powered transtibial prostheses integrated with sensory and motor nerve fibers or muscles via a percutaneous pylon have the great potential for improving quality of prosthetic locomotion as discussed above, there have been no rigorous studies on animal models that tested the feasibility and performance of such prostheses. Prior to implementing this technology in people with limb loss, preclinical animal studies should address the following important questions: (i) Do these prostheses improve symmetry of locomotion and to what extent? (ii) How does continual electrical stimulation of peripheral nerves affect the nerve structural integrity and function? (iii) Does stimulation of sensory nerves engage proper reflex responses and how they change over time? (iv) Do residual muscles and their myoelectric activity controlling prosthetic actuators degrade over time to a degree that cannot be compensated by the control system? (v) Does the porous titanium implant serving as a prosthesis-body gateway allow for skin ingrowth and reduction of the infection rate, etc.

The use of animal models for testing sensing, powered prostheses during locomotion may be challenging. The first challenge is securing a limb prosthesis on the animal. Rodents, cats, and dogs are notorious for removing externally attached assistive devices (Mich, 2014); therefore, the use of bone-anchored implants for prosthesis attachment appears a viable option (Fitzpatrick et al., 2011; Farrell et al., 2014b; Jarrell et al., 2018). Another challenge is strict limitations on prosthesis small size and mass and a relatively high power output. For the cat of 3 to 4 kg, for example, the half of tibia length is approximately 55 mm (Klishko et al., 2014); mass of the foot with half of the shank is ∼80 g (Hoy and Zernicke, 1985); the average peak of the ankle moment during level walking is 0.73–0.75 Nm (Gregor et al., 2006; Prilutsky et al., 2011); and the average peak of ankle positive power in the same conditions is 0.86 W (Prilutsky et al., 2011). Thus, each component of the prosthesis [prosthetic foot, sensors, actuator, battery, neural stimulator and amplifier, microprocessor unit (MCU), and electronics] should be carefully selected to satisfy these requirements. ABS plastic, carbon fiber, or fiberglass are lightweight materials with high ultimate strength and can be used for prosthetic foot fabrication (Delussu et al., 2013; Farrell et al., 2014b; Corbett et al., 2018). Options for appropriate prosthetic actuators and batteries are more limited as they need to satisfy the conflicting requirements for lightweight and high power output. Soft pneumatic actuators, satisfying the above requirements, have been recently developed and used in limb prosthetic and orthotic applications in people and animals (Ferris et al., 2005; Roche et al., 2014; Florez et al., 2017). However, these actuators require large off-board air pressure regulators and therefore are better suited for rehabilitation and research of assisted locomotion on a treadmill. Linear electromechanical actuators has demonstrated sufficient power production in relatively light wearable, powered prosthetic ankles during human walking (Blaya and Herr, 2004; Garcia et al., 2011; Realmuto et al., 2011). Considering the above limitations on size, weight, and moment production for the cat prosthetic ankle, a miniature linear actuator (PQ12-63-06-P, Actuonix, BC, Canada) appears to be a good choice. With its small weight of 15 g, stroke length of 20 mm, and maximum force of 45 N, it should produce an extension ankle moment of ∼1 Nm with the moment arm of ∼0.025 m corresponding to that of the cat Achilles tendon (Prilutsky et al., 1996). A further challenge is the selection of an appropriate feedback control law for the prosthesis. Although a wide variety of feedback control laws are employed by terrestrial animals including humans during locomotion (Edwards and Prilutsky, 2017), proportional-derivative control laws are often used in orthotic-prosthetic ankle emulators controlled by powerful off-board electric motors or pressure regulators to reproduce either the desired joint moment or joint position (Sawicki and Ferris, 2009; Caputo and Collins, 2014). In wearable powered prostheses, finite-state controllers are often used that do not require exact tracking of a desired joint moment or angular trajectory (Au et al., 2007; Shultz et al., 2016) and thus permit the use of lighter and less powerful actuators.

The goal of this work was to develop and benchtop characterize a prototype of a bone-anchored, powered, and sensing transtibial prosthesis for a feline animal model of prosthetic gait. The developed prototype included an ABS plastic foot with force sensor, stimulator of a sensory nerve, EMG amplifier, linear actuator, battery, and microprocessor. The prototype satisfied the design criteria for prosthesis weight and moment production. In benchtop testing, the performance of a finite-state control scheme for the prosthesis was evaluated by subjecting the prosthesis to rhythmic loading that simulated the stance and swing phases of locomotion. A force sensor on the ground detected two motion states – the stance and swing, and the linear actuator generated an extension and flexion moment, respectively. An empirical relationship between muscle activity and ankle moment developed using our previous data were simplified by a step function with a variable gain. The gain of the extension moment was adjusted in each cycle automatically via a wireless interface and off-board PC to reduce the error between the desired peak of the ground reaction force (GRF) and the measured peak. The prosthetic prototype was able to reproduce the desirable GRF peaks within several cycles.

## Materials and Methods

### Prosthesis Design

#### Prosthesis Components

**FIGURE 1:**
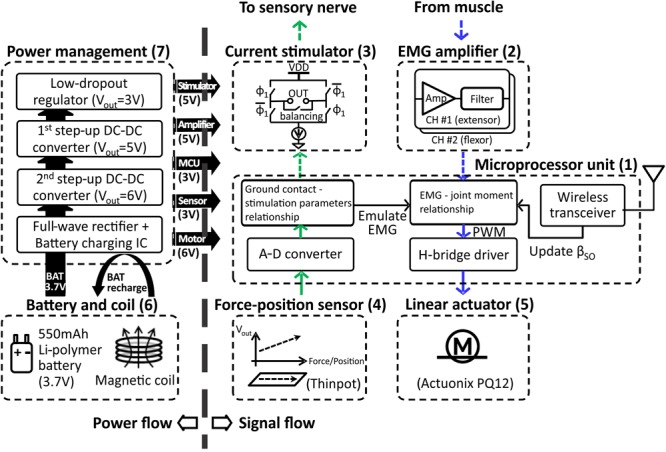
Block diagram illustrating power flow (on left) and signal flow (on right) between prosthetic components. The sensory pathway of signal flow on right is indicated by the green arrows; the motor pathway is indicated by blue arrows. The pathways represented by dashed arrows are not implemented in the prosthetic prototype, but will be implemented when the prosthesis is worn by the cat. See text for details.

The prosthesis comprised (1) MCU, (2) EMG amplifier, (3) current stimulator, (4) force-position sensor, (5) linear actuator, (6) battery and coil, (7) power management, and (8) prosthetic foot. (1) The MCU model CC2510F32 (Texas Instruments, TX, United States) included 8051 microcontroller and wireless transceiver with low-power consumption. (2) EMG amplifier INA128 with gain of 1000 (V/V) (Texas Instruments, TX, United States) included an embedded Sallen-Key active band-pass filter to suppress both motion artifact and ambient noise. (3) Current stimulator had discrete n-type field effect transistors (nFETs) and p-type field effect transistors (pFETs) designed to generate biphasic current pulses, while a programmable resistor AD5162 (Analog Devices, MA, United States) adjusted the current level of the pulses using current steering. The stimulator was tested in walking cats – electrical stimulation was applied to the distal tibial nerve during the stance phase of walking and reduced or reversed effects of paw pad anesthesia on the duty factor and step length symmetry (Park et al., 2015, 2016). (4) ThinPot linear force-position sensor (Spectra Symbol, UT, United States) was fixed on the bottom of the J-shaped foot, between the J-shaped plastic foot and the rubber layer. The sensor can record normal force with the 1-bit resolution at a threshold of 0.7 N. This is sufficient to detect paw contact during walking in cats (Park et al., 2015, 2016). (5) A miniature linear actuator PQ12-63-06-P (Actuonix, BC, Canada) with a brushed DC motor and transmission gear with a 63:1 ratio can produce a 20-mm stroke, which corresponds approximately to muscle-tendon unit length changes of a cat ankle extensor (soleus, SO) during locomotion (Gregor et al., 2006). This single linear actuator with an H-bridge motor driver (DRV8837, Texas Instruments, TX, United States) could extend and flex the prosthetic joint and thus reproduce actions of the ankle extensors (e.g., SO) and flexors (e.g., tibialis anterior, TA). (6) A Li-polymer rechargeable battery GM053040 (550 mAh, 5 mm × 30 mm × 40 mm) was selected as the power source. Its maximum discharge current (550 mA) corresponds to the maximum stall current of the linear actuator PQ12-63-06-P. We estimated the battery would last before recharging for 1.5 h based on current requirements of the linear actuator to generate force of 20 N (∼200 mA), current requirements for other electronic components (<20 mA), the DC–DC conversion ratio (∼2:1) and efficiency (∼85%), and walking duty cycle (<75%). The inductive coil was provided for wireless recharging. (7) Power management generated 3V outputs for the MCU and foot force-position sensor, 5 V outputs for the EMG amplifier and current stimulator, and a 6 V output for the linear actuator. (8) J-shaped foot was 3D printed from the ABS plastic capable of withstanding forces of 60–90 N that exceed peak ground reaction forces (GRF) during cat walking by two to three times (Corbett et al., 2018). The diagram in **Figure 1** illustrates the signal and power flow between the prosthetic components. The signal flow from the ThinPot linear force-position sensor on the foot (4) to the current stimulator (3) represents the sensory pathway (green arrows), whereas the signal flow from the EMG amplifier (2) to the linear actuator (5) – the motor pathway (blue arrows).

**FIGURE 2:**
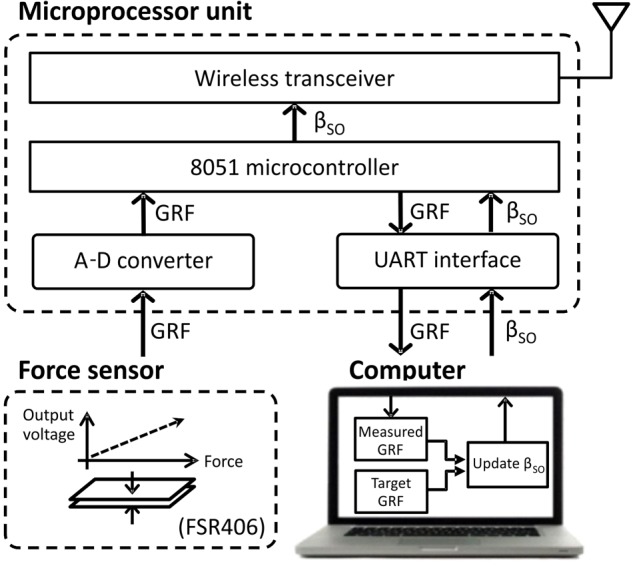
Block diagram of external devices (computer, ground force sensor, and microprocessor unit) that update the extension gain β_SO_, based on the ground reaction force (GRF) peak, and transmit it wirelessly to the prosthesis. See text for details.

The prosthesis was wirelessly connected with external devices, i.e., a force sensing resistor FSR406 (Interlink Electronics, CA, United States) mounted on the floor and a computer monitoring GRF and adjusting a motor gain of the linear actuator in real time. An external MCU with a wireless transceiver and microcontroller provided communications between the external devices (**Figure 2**) and the prosthesis.

#### Prosthesis Assembly

**FIGURE 3:**
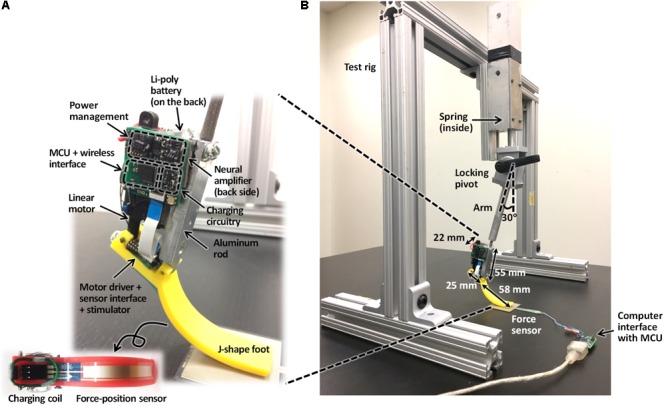
Prosthesis prototype **(A)** and test rig with the attached prosthesis **(B)**.

A rectangular aluminum bar (6061-T6511, Metalsdepot, KY, United States) 55 mm in length served as a structural frame for the prosthesis (**Figure 3**). The bar was connected to the J-shaped plastic foot via a pivot. The aluminum bar was also connected to the percutaneous pylon that would be implanted into the medullary cavity of the cat tibia and interfaced with residual cutaneous nerves and SO and TA muscles via implanted electrodes.

The linear actuator (see above) was attached to a posterior side of the aluminum bar at a 25-mm distance from the ankle pivot (this distance approximately corresponds to the moment arm of the cat Achilles tendon with respect to the ankle (Goslow et al., 1973; Prilutsky et al., 1996). Two separate printed circuit boards (PCBs) were placed to the right of the linear actuator and the flat part of the J-shaped foot. The MCU, wireless interface, EMG amplifier, and power management integrated with the PCB were placed to the right of the linear actuator. The motor driver, sensor interface, and stimulator integrated with the PCB were fixed on the flat part of J-shaped foot. Finally, a Li-polymer rechargeable battery was mounted to the left of the linear actuator.

The prosthesis components were selected to satisfy the design criteria for prosthesis weight and moment production. As a result, the prosthesis mass was 63 g with the maximum available moment (stall moment) of 1 Nm. The stall moment was calculated from the maximum push/pull force of the linear actuator (40 N) and the actuator moment arm with respect to the pivot (note that we measured the maximum force of the linear actuator and the obtained value of 40 N was slightly lower that 45 N reported by manufacturer). The value of 1 Nm is close to the maximum ankle moment during level walking in the cat (McFadyen et al., 1999; Gregor et al., 2006; Prilutsky et al., 2011).

### Prosthesis Control

#### Finite-State Controller

**FIGURE 4:**
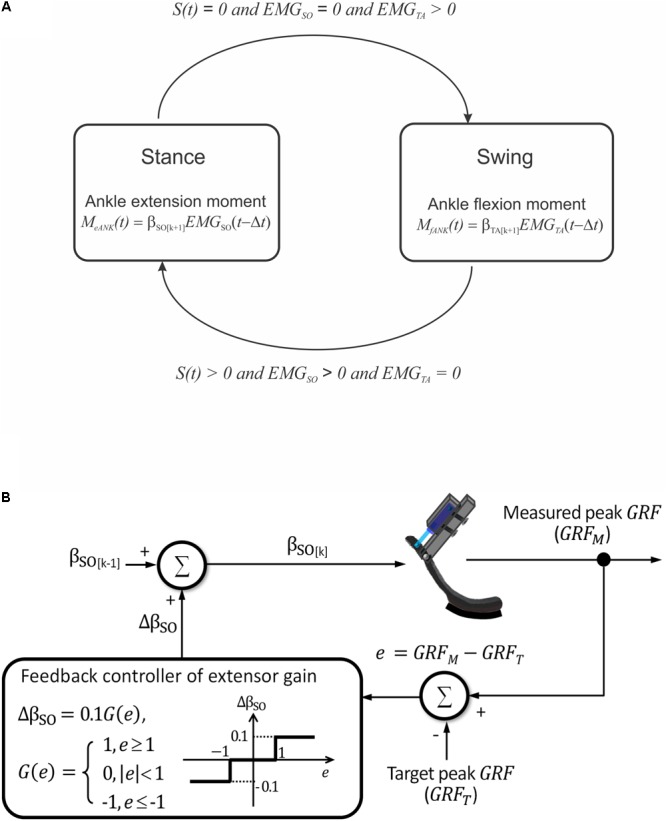
Schematics illustrating prosthesis control. **(A)** Two finite states of prosthesis loading and conditions for transitions between them. *S*(*t*) is a step function indicating presence [*S(t)* > 0] or absence [*S(t)* = 0] of ground contact. *EMG_SO_* and *EMG_TA_* are step functions indicating presence or absence of EMG activity in ankle extensor soleus (SO) and ankle flexor tibialis anterior (TA), respectively. **(B)** Closed-loop modulation of extension gain b_SO_. The gain is changed by 10% in each cycle *k* depending on the value of error *e* between the measured and target peaks of ground reaction force (GRF).

A simple finite-state machine controller was implemented to control the linear actuator (**Figure 4A**). Transitions between the two states – stance and swing – depended on the presence of contact with the ground and EMG activity of a residual ankle extensor and flexor muscles. Transition from the stance to swing state was triggered by (i) foot unloading (interruption of contact with the ground), (ii) terminating EMG activity of the ankle extensor, and (iii) initiating EMG activity of the ankle flexor (**Figure 4A**). These three conditions triggered a pushing stroke of the linear actuator leading to a flexor moment at the ankle. Transition from the swing to stance state was initiated by (i) onset of ground contact with the foot, (ii) onset of EMG activity of the ankle extensor, and (iii) offset of EMG activity of the ankle flexor. These conditions triggered a pulling stroke of the linear actuator producing an extension ankle moment.

#### Ankle Moment–EMG Relationship

To modulate the output of the linear actuator during the stance and swing states of walking, we established a relationship between EMG activities recorded from ankle extensor and flexor muscles and the resultant ankle moment (motor pathway, **Figure 1**).

The relationship between EMG activity of an ankle extensor SO and ankle flexor TA and ankle moment during level walking in the cat was obtained from previously recorded EMGs and ankle moment (Prilutsky et al., 2011; Markin et al., 2012) using a multivariate linear regression analysis in software STATISTICA 7 (StatSoft, United States). The equation had the following form (Prilutsky et al., 2005):

(1) $$\begin{array}{c} M_{ANK}(t)=\beta_{0}+\beta_{SO}EMG_{SO}(t-\Delta t)+\beta_{TA}EMG_{TA}(t-\Delta t), \end{array}$$

where M_ANK_ is the ankle joint moment in Nm; *EMG_SO_* and *EMG_TA_* are normalized EMG activities of SO and TA muscles, changing from 0 to 1; *t* is time and Δ*t* ≈ 60 ms is the electromechanical delay between the appearance of EMG activity and the onset of the resultant joint moment (Gregor et al., 2006); β_o_ ≈ 0 (see Results), β_SO_ and β_TA_ are empirical constants (measured in Nm). Approximately two-thirds of total 22 walking cycles (*n* = 15) from three cats were randomly selected and used to derive regression equation (1). The remaining cycles (*n* = 7) were used to compare the predicted ankle moment M_ANK_ with the experimental one. The detailed description of how the joint moments and EMG activities were obtained and processed can be found in the original publications (Prilutsky et al., 2005, 2011; Markin et al., 2012).

#### Ground Contact Pressure and Tactile Perception

In our preliminary studies (Park et al., 2015, 2016), we have established the relationship between output of the force-position sensor under the cat hindpaw and electrical stimulation of the distal tibial nerve (sensory pathway, **Figure 1**) that apparently perceived by the cat as contact with the ground during walking. When the output of the force sensor exceeded a threshold (indicating the stance phase), the current stimulator delivered stimulation (trains of 200-μs biphasic rectangular pulses, 100 Hz, 1.2 T) to the distal tibial nerve. This sensory nerve stimulation reduced or reversed effects of local anesthesia of the ipsilateral hind- and forepaws on the step length symmetry and duty factor (Park et al., 2015, 2016).

#### Implementation of Control During Benchtop Testing

For benchtop testing of the developed prosthesis outside the animal in this study, both the sensory and motor pathways were simplified. The simplified sensory pathway transmitted information about the timing of ground contact, measured by the force-position sensor on the foot, to the linear actuator instead of the current stimulator (**Figure 1**). The timing of ground contact was described as a unit step function *S*(*t*):

(2) $$\begin{array}{c} {S(t)=H(F(t)-F}_{\mathrm{TH}}), \end{array}$$

where F(t) is the recorded force-position sensor output, F_TH_ is the force detection threshold, and *H*(*x*) is a Heaviside step function, i.e., *H*(*x*) = 1 if *x* > 0 and *H*(*x*) = 0 if *x* ≤ 0. Function *S*(*t*) defined the stance and swing phases (finite states of the system; **Figure 4A**), and this phase information was used to emulate a simplified motor pathway, i.e., the relationship between the ankle moment and EMG of SO and TA muscles. Specifically, EMG activity of SO and TA muscles was emulated by unit step functions representing the timing of muscle activity derived from the ground contact information. SO EMG was computed as

(3) $$\begin{array}{c} EMG_{SO}(t)=S(t-\Delta t_{SO})-S(t-(\Delta t_{SO}+T_{SO})), \end{array}$$

where Δt_SO_ is the phase delay between the previous stance phase offset and subsequent SO EMG onset, *T_SO_* is the duration of EMG_SO_ activity, *S(t)* is the step function representing contact information (see Eq. 2). In the tests described here, the following parameters of Eq. 3 were used (Prilutsky et al., 2005, 2011; Markin et al., 2012): Δt_SO_ = 100 ms and *T_SO_* = 500 ms.

TA EMG activity was computed as

(4) $$\begin{array}{c} EMG_{TA}(t)=S(t-\Delta t_{TA})-S(t-(\Delta t_{TA\;}+T_{TA})) \end{array}$$

where Δt_TA_ is the phase delay between the previous stance phase onset and subsequent TA EMG onset, *T_TA_* is the duration of EMG_TA_ activity; Δt_TA_ = 400 ms and *T_TA_* = 200 ms (Prilutsky et al., 2005, 2011; Markin et al., 2012).

The emulated EMG signals (Eqs 3 and 4) were used to control the linear actuator with a dual polarity. The ankle joint moment was calculated using Eq. 1 and emulated EMG activity of SO and TA obtained from Eqs 3 and 4 (**Figure 4A**). Because SO and TA during walking have reciprocal activity and β_0_ is close to zero (see Eq. 1 and Results), calculations of the ankle extension and flexion moments were simplified as M_e ANK_(t) = β_SO_ EMG_SO_(t - Δt), and M_f ANK_(t) = β_TA_ EMG_TA_(t - Δt), respectively (see **Figure 4A**). In these equations, β_SO_ and β_TA_ are extension and flexion motor gains.

#### Closed-Loop Updates of Extension Motor Gain

The maximum of extension gain β_SO_ was set at 1 Nm. The updated value of the gain in a next cycle could be increased or decreased by 10% depending on the difference *e* = *GRF_M_* -*GRF_T_* between the measured GRF peak (*GRF_M_*) and a target GRF peak (*GRF_T_*), respectively, in the current cycle (**Figure 4B**):

(5) βSO[K]  = βSO[K-1] +0.1 G(e) 

where *k* is the cycle number and G(e) = 1 if e ≥ 1, G(e) = 0 if |e| < 1 , G(e) = -1 if e ≤-1. Thus, if the *GRF_M_* exceeded or was less than the target value by 1 N or more, the current extension gain would be decreased or increased by 10%, respectively; otherwise, the gain would not change (**Figure 4B**).

### Benchtop Characterization of Prosthesis

During the benchtop characterization, we imposed rhythmic loading on the prosthesis to simulate the stance and swing phases of walking and to test the finite-state machine controller (**Figure 4A**) with a closed-loop modulation of the extension gain in real time (**Figure 4B**).

#### Design of a Test Rig

To perform benchtop characterization, we designed a test rig made of aluminum bars with L-shaped connectors, a zinc-plated compression spring, locking pivot, and prosthesis support arm (**Figure 3B**). The force produced by the compressed spring, along with the weight of the prosthesis and its support arm, caused loading of the prosthesis during contact with the ground that was comparable to GRF exerted by the hindpaw during normal level walking in the cat. The support arm was set at a vertical angle of 30° so that the J-shaped prosthetic foot could be in contact with the ground starting at both full flexion (at foot contact) until full extension (foot off) of the ankle joint.

#### Test Procedure

Each test cycle started from onset of the swing state of the controller – the prosthesis foot was positioned just above the ground, prosthetic ankle was fully extended, and the linear actuator started producing a flexion ankle moment. This prosthesis position corresponded to full relaxation of the compression spring. The researcher raised the prosthesis by the hand to a height of ∼40 mm, at which the spring was fully compressed, and then the prosthesis was released. The fully compressed spring accelerated the prosthesis toward the ground vertically. Given spring deformation of ∼40 mm and stiffness of 0.36 N/mm, the spring applied ∼14 N to the prosthesis when it was released by the hand.

When the prosthesis touched the ground, the foot force-position sensor detected the ground contact and the conditions for the swing to stance state transition were satisfied: *S(t)* > 0 (Eq. 2), *EMG_SO_* > 0 (Eq. 3), and *EMG_TA_* = 0 (Eq. 4). At that instant, the linear motor initiated a pull stroke and generated extension ankle moment (*M_eANK_*, see **Figure 4A**). When the prosthetic joint reached the maximum extension at the end of stance phase, the prosthesis was lifted by the experimenter’s hand and raised against the compression spring as described above. As soon as ground contact was lost, the conditions for the stance to swing state transition were satisfied: *S*(*t*) = 0 (Eq. 2), *EMG_SO_* = 0 (Eq. 3), and *EMG_TA_* > 0 (Eq. 4). At that instant, a flexion ankle moment was generated (*M_fANK,_* see **Figure 4A**), and the prosthesis joint angle returned to the fully flexed position. Cadence of prosthesis loading in these tests corresponded to a typical cadence of walking cats (Gregor et al., 2006).

We also tested the ability of the feedback controller to modulate the extension gain *β_SO_* and thus the magnitude of the exerted ankle moment (*M_eANK_*, **Figures 4A,B**) in real-time. The produced peak GRF (*GRF_M_*) was measured by the force sensor FSR406, mounted on the ground under the prosthesis foot (**Figure 3B**). The target value of *GRF_T_* was set and compared with the *GRF_M_* value in a custom designed LabView (National Instruments, TX, United States) application on the off-board computer. Based on the operating principle of the DC motor, we assumed that the extension gain *β_SO_* (and thus extension moment *M_eANK_*) was proportional to the duty cycle of pulse-width modulation (PWM) of control signal (Weber, 1965). The maximum value of extension gain (*β_SO_* = 1 Nm) corresponded to the extension ankle moment *M_eANK_* = 1 Nm and PWM = 100%. With this maximum gain, the linear actuator produced the maximum force of 40 N and could generate the maximum ground reaction peak of ∼13–15 N (see Results). The extension gain *β_SO_* (corresponding to PWM) was updated in each test cycle based on Eq. 5 (**Figure 4B**). The closed-loop control system was tested at three target values of *GRF_T_*: 14, 6, and 12 N. These three target forces were pre-programmed in the microprocessor to occur at the onset of testing, at the end of cycle 2 and at the end of cycle 8, respectively. During testing, the flexion gain *β_TA_* was set at the maximum value of -1 Nm and not changed (**Figure 4A**).

## Results

### Ankle Moment-EMG Relationship

**FIGURE 5:**
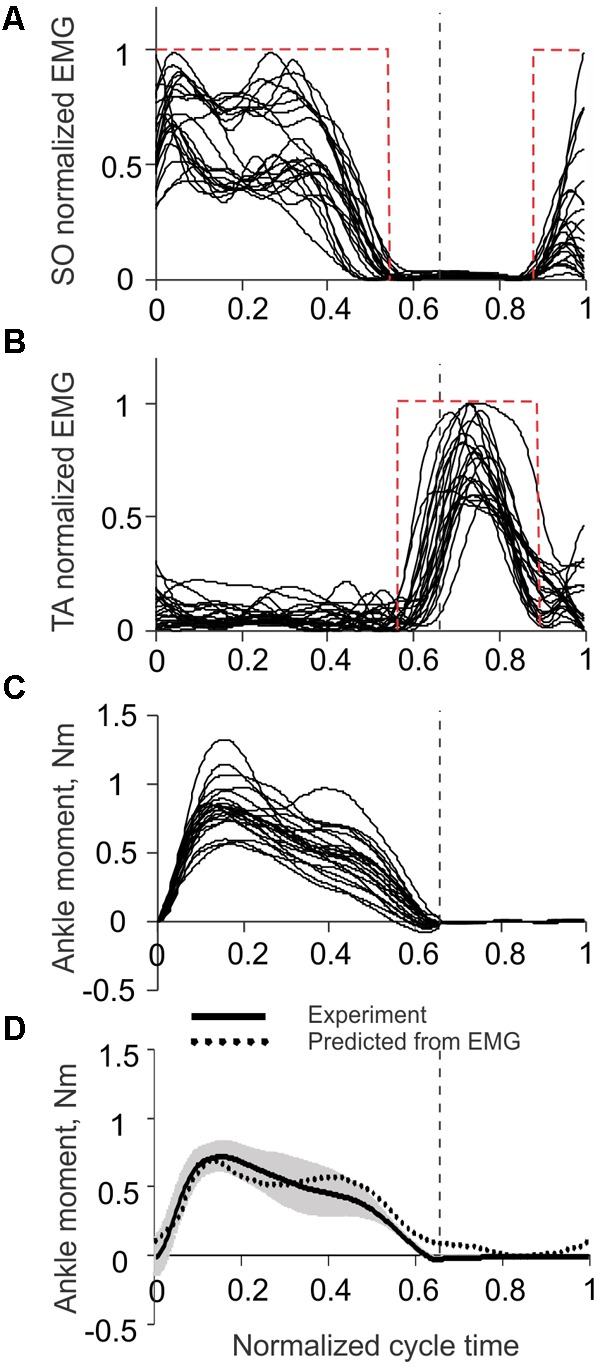
Ankle joint moment and EMG activity of soleus (SO) and tibialis anterior (TA) muscles during level walking in the cat. The vertical dashed lines separate the stance and swing phases. Experimental EMG and ankle moment data are taken from 22 cycles of level walking of three cats (mass 3.55 ± 0.65 kg; mean ± SD) (Prilutsky et al., 2011; Markin et al., 2012). **(A)** Normalized SO EMG during level walking in the cat. The red dashed rectangle corresponds to emulated SO EMG signal (see text for details). **(B)** Normalized TA EMG during level walking in the cat. The red dashed rectangle corresponds to emulated TA EMG signal (see text for details). **(C)** Ankle joint moment during level walking in the cat; positive values correspond to extension (plantar flexion). **(D)** Ankle moment obtained experimentally (solid line with gray shade, mean ± SD) and predicted from SO and TA EMGs using Eq. 1 (dotted line). Positive values correspond to extension (plantar flexion).

Rectified and low-pass filtered EMG activities of SO and TA, as well as the corresponding ankle joint moments, recorded in Prilutsky et al. (2011), Markin et al. (2012) during 22 cycles of level walking in three cats (**Figures 5A–C**), were used to obtain the regression Eq. 1. The empirical constants in Eq. 1 were β_0_ = 0.023528 Nm, β_S0_ = 0.969663 Nm, and β_TA_ = 0.052416 Nm. The coefficient of multiple correlation for Eq. 1 was *r* = 0.874 (*p* < 0.05). The ankle moment as a function of the normalized cycle time computed from SO and TA EMGs using Eq. 1 was generally within one standard deviation from the mean experimental moment (**Figure 5D**). As explained in Materials and Methods, SO and TA EMG activity was simplified for the purpose of the benchtop testing of the prosthesis by step functions *EMG_SO_* and *EMG_TA_* (Eqs 3 and 4). These step functions are shown in **Figures 5A,B** by red dashed rectangles.

### Finite State Controller With Closed-Loop Update of Extension Gain

**FIGURE 6:**
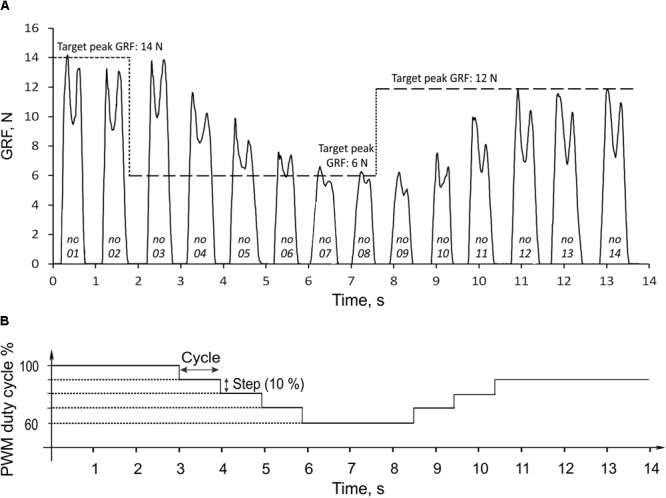
Ground reaction force (GRF) during 14 cycles of prosthesis loading **(A)** and the corresponding changes in PWM duty cycle of the actuator **(B)**. The three target values of GRF peak (14, 6, and 12 N) are indicated by the horizontal dashed lines in **(A)**. The numbers at bottom of force traces indicate the cycle number. For details, see text.

During rhythmic loading of the prosthesis, the finite state controller correctly identified the stance and swing states based on the signal from the force-position sensor on the bottom of the foot. The linear actuator produced pulling strokes (extension ankle moments) in the stance state and pushing strokes (flexion moments) in the swing state. In the example in **Figure 6A**, the prosthesis produced GRF in 14 cycles of rhythmic loading; the corresponding changes in the PWM duty cycle are shown in **Figure 6B**. In the first two cycles, the target GRF force was 14 N, which corresponded to the maximum capacity of the linear actuator (PWM duty cycle was 100%). Since the GRF peaks produced in these cycles were within ±1 N of the target value, the extension gain *β_SO_*, and PWM were not changed (**Figure 6B**, Eq. 5). At the end of stance phase of cycle 2, when the target force was reduced from 14 to 6 N, the force error *e* (Eq. 5) was detected in stance of cycle 3 and the extension gain *β_SO_*, and PWM were reduced by the control system by 10% in cycles 4 through 6 until the peak GRF error during stance became smaller than 1 N in cycle 7 (**Figure 6**). The peaks of GRF in cycles 7 and 8 were maintained near the target force of 6 N within ±1 N, and no changes in PWM occurred. After the target force changed at the end of cycle 8 from 6 to 12 N, the controller detected the force difference *e* in stance of cycle 9 and increased PWM by 10% in cycle 10. Since the measured GRF peaks in cycles 10 and 11 were lower than the target value, PWD was increased again by 10% in cycles 11 and 12. Since the GRF peaks in cycles 13 and 14 were within ±1 N from the target value of 12 N, no changes in PWD occurred in these cycles (**Figure 6**).

The peak GRF values during the transition period from the target change to achieving the target by the system (cycles 3 through 7 and 8 through 12; **Figure 6A**) could be considered the system step response to the error *e* input (**Figure 6B**). In the current control system design, the response time corresponded to the duration of one cycle. The prosthesis closely reproduced the target GRF peaks in steady state cycles 1–3, 7–9, and 12–14 (**Figure 6A**). The absolute error of peak GRF across all three target values was 0.31 ± 0.23 N (mean ± SD), and the relative error (absolute error normalized to the target value) was 3.49 ± 3.06%.

### Ground Reaction Forces Produced by the Prosthesis

**FIGURE 7:**
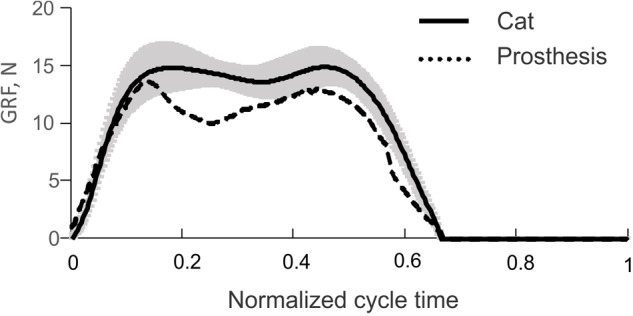
Vertical ground reaction force (GRF) recorded during level walking in the cat (solid line and shade, mean ± SD; computed using data from Prilutsky et al., 2011) and mean GRF recorded during the first three cycles of prosthesis testing (dashed line), see **Figure 6A**.

The time profiles of GRF measured under the prosthetic foot in 14 consecutive cycles had a double-peak pattern (**Figure 6A**). The mean GRF peak in cycles 1 through 3, where the PWM duty cycle was set to 100% to produce maximal GRF peaks, was 13.8 ± 0.5 N. This value was within one standard deviation of the GRF mean peak (14.9 ± 1.6 N) obtained in walking cats (**Figure 7**).

The comparison of the prosthetic GRF profiles averaged across cycles 1 through 3 with the experimental GRF recorded previously during level walking in cats (Prilutsky et al., 2011) – the same 22 cycles from which ankle moments and EMG patterns in **Figure 5** were obtained, demonstrated close qualitative and quantitative agreements (**Figure 7**). Specifically, both patterns had two peaks – one in the early stance phase (leg contact) and the other one in the late stance phase.

## Discussion

We developed a powered, sensing transtibial prosthesis for the use in the feline animal model of prosthetic gait. This animal model is needed for testing feasibility and performance of bone-anchored limb prostheses integrated with residual sensory nerves and muscles during locomotion (see Introduction). The size, mass, and maximum extension moment of the prosthesis closely matched the corresponding parameters of the cat foot-ankle with the distal shank and the peak ankle extension moment produced during level walking in the cat (Gregor et al., 2006, 2018; Prilutsky et al., 2011). The prosthetic powered ankle joint was designed for control of the linear actuator by the recorded EMG activity of the residual ankle extensor and flexor muscles. The ability of the prosthesis to detect timing of ground contact will allow for delivering tactile sensory feedback by phase dependent stimulation of sensory nerves. The foot force-position sensor detecting touch with the ground in this study was used in the past to trigger electrical stimulation of the distal tibial nerve during the stance phase of walking and to provide tactile feedback to the nervous system of walking cats with the anesthetized hindpaw (Park et al., 2015, 2016).

In the present benchtop testing of the prosthesis, only selected prosthesis functions were characterized. They included detecting timing of ground contact onset and offset, control of transitions between the stance and swing states by the finite-state machine controller, and a real-time automatic modulation of the extension gain based on the measured GRF peak in each loading cycle (**Figure 4**). The results of testing demonstrated that the prosthesis was able to produce the extension and flexion ankle moments in the appropriate loading states. The prosthesis was also able to generate appropriate GRF peaks by modulating the extension gain in a closed-loop real time control. In addition, the prosthesis was capable of generating realistic GRF forces similar to those observed during normal level walking in the cat. Although the maximum GRF peaks were slightly lower than the desired value of 15 N (a typical GRF peak during level walking in the cat) and much lower than peak forces during 27°-upslope walking (17-22 N; Gregor et al., 2006; Prilutsky et al., 2011), we expect that proximal joints may be able to compensate for this difference during cat walking with the powered prosthesis. This expectation is based on a recent study demonstrating that cats walking with a passive bone-anchored transtibial prosthesis with no active ankle extension are able to generated ∼70 and ∼50% of the normal GRF peak observed in intact level and 27°-upslope walking, respectively (Jarrell et al., 2018).

The double-peak GRF profiles generated by the prosthesis (**Figure 6**) were not expected because the control system was designed to reproduce just a target GRF peak. It appears that the observed GRF profile is a result of interactions between the constant moment produced by the linear actuator and passive dynamics of the prosthesis and its support system. The two GRF peaks had different magnitudes, and the second peak was lower than the first (**Figure 6**).

The magnitude of the second peak of vertical GRF depends on the magnitude of ankle extension moment in the late-stance phase of prosthetic walking in humans. For example, reduction in passive foot stiffness leads to a parallel decrease in the second GRF peak and ankle extension moment peak (Fey et al., 2011). The use of powered ankle prostheses decreases or eliminates the differences in second GRF peak and ankle extension moment magnitude between the intact and prosthetic limbs in humans (Rabago et al., 2016; Shultz et al., 2016). Since our powered prosthesis with its control system is designed to maintain a target GRF peak, we do not expect a close match of the generated GRF profile with that of the intact animal. This expected mismatch should not necessarily lead to asymmetric walking unless there is a substantial mismatch in the GRF impulse.

The linear actuator PQ12-63-06-P was selected for the cat transtibial prosthesis because it satisfied strict limitations on the size and mass of the cat foot-ankle and distal shank. To maximize the force output of the actuator to ensure it could produce its maximum moment of 1 Nm, we increased its duty cycle from its optimum value of 20%, recommended by the manufacturer as the most efficient, to 100%. We verified consistency of the actuator operation with the duty cycle of 100% over multiple cycles in our benchtop prosthesis testing. We found that this linear actuator at the duty cycle of 100% could generate consistent levels of GRF for over 100 cycles. This number of cycles is sufficient for a single recording session in the cat.

It may be necessary to increase the moment arm of the linear actuator with respect to ankle joint or replace this actuator with a larger one if testing in the animal would demonstrate its inability to generate sufficient ankle moment and power. However, a larger size of the actuator and battery would increase demands on the knee and hip flexor muscles during the swing phase of walking and could lead to abnormal asymmetric locomotor pattern.

In our benchtop testing of the prosthesis prototype, the force sensing resistor FSR406 mounted on the floor (**Figure 3B**) measured vertical GRF peaks, and the linear force-position sensor (ThinPot) attached to the bottom of the foot (**Figure 3A**) detected ground contact timing used to emulate extensor and flexor EMG bursts and determined onset-offset times of the linear actuator (**Figure 4A**). In the actual implementation of the prosthesis in the animal, we plan to mount the force-sensing resistor FSR406 or a similar one on the bottom of the prosthetic foot to serve both functions, i.e., detecting ground contact and measuring GRF peaks. In that case, wireless communication between the prosthesis and external computer will be used to monitor, modify, and record characteristics of the control system (target GRF peaks, actuator gains, stimulation parameters, EMG, etc.).

One potential limitation of the force-sensing resistor FSR406 for monitoring the peak GRF is that it can only measure the normal component of the 3D GRF vector (vertical component in this study, **Figures 6A, 7**), although the other two GRF components are also important for accurate description of foot interaction with the ground (Aubin et al., 2008). During level cat walking, the normal peak GRF force exceeds the anterior-posterior and medial-lateral peaks by ∼5 and >10 times, respectively (Farrell et al., 2014a). Thus, the peak of the normal GRG component might still be used to monitor and modify the prosthesis output during level walking in the cat. However, during 27°-upslope cat walking the normal and tangential (in progression direction) peaks are comparable (Gregor et al., 2006, 2018; Prilutsky et al., 2011). Therefore, for this walking condition some modifications in the GRF target or control algorithm may be necessary.

In the animal testing, the GRF peak measured by the force sensor FSR406 on the foot in each walking cycle will be compared with a preset target value, and gains *β_SO_* and *β_TA_* will be changed in real time if necessary. Information about ground contact onset and offset determined by the same sensor will be used to control timing of electrical stimulation of the sensory nerves. We could use the timing of ground contact to control the linear actuator as demonstrated in this study. However, we plan to use recorded EMG signals from residual SO and TA to estimate the ankle moment (Eq. 1) and use either the estimated moment peak or moment profile for control of the linear actuator. Gains *β_SO_* and *β_TA_* could be modified based on the measured GRF peaks (**Figure 4B**) or/and predicted ankle moment peak. This type of control seems more intuitive for the user (Ortiz-Catalan et al., 2014; Kannape and Herr, 2016) since it includes a highly adaptive living system in the control of the prosthesis output.

In our planned animal studies, we will evaluate the contribution of sensory nerve stimulation to SO and TA EMG activity magnitude, to symmetry of walking and to other locomotor characteristics by comparing walking with and without phase dependent stimulation of sensory nerves. Changes in quality of EMG signals and in activation thresholds of sensory nerves [recorded action potentials in the sciatic nerve in response to stimulation of the distal tibial, sural or superficial peroneal nerves while the animal is sedated (Ollivier-Lanvin et al., 2011; Park et al., 2016)] will be determined over several months. During testing the prosthesis in the animal model, we plan to add another sensory feedback signal – contact force from the dorsal surface of the prosthetic foot. Another force sensor FSR406 will detect contact of the dorsal surface of the prosthetic foot with an external object and trigger electrical stimulation of the superficial peroneal nerve if the contact occurs in the swing phase of locomotion. The superficial peroneal nerve is a cutaneous nerve innervating skin on the dorsum of the foot (Crouch, 1969). Electrical stimulation of this nerve during swing elicits stumbling corrective response in the cat (Forssberg, 1979; Wand et al., 1980; Quevedo et al., 2005), which helps the animal avoid tripping by enhancing stepping over the obstacle.

In the end of the study, the animals will be euthanized and the residual limb with the porous titanium implant, residual muscles, and nerves with implanted electrodes will be harvested for histological analysis (Farrell et al., 2014b,c). This analysis will reveal the extent of skin and bone ingrowth into the percutaneous implant and integrity of implanted muscles and nerves. The results of our planned animal studies will inform future designs of transtibial prostheses integrated with the residual limb in people.

## Conclusion

In conclusion, the designed prototype of a feline bone-anchored, sensing, powered transtibial prosthesis demonstrated the ability to reproduce values and patterns of the GRF observed during normal walking in the cat. The prosthesis dimensions, mass, and extension moment produced were similar to the corresponding characteristics of the cat. The prosthesis was designed for use with a porous titanium pylon implanted in tibia (Pitkin et al., 2012; Farrell et al., 2014b; Jarrell et al., 2018) that could serve as a gateway for transmission of feedback (from the prosthesis to the peripheral sensory nerves) and feedforward (from implanted muscle electrodes to the prosthetic actuator) signals between the prosthesis and the residual limb.

## Author Contributions

BP and SD did conception and design of research; HP, MI, BP, and SD did prosthesis design; HP, MG, AK, and PB did design of control algorithm; HP and MI did experimental recordings; HP analyzed data; HP, AK, and PB prepared figures. HP, MG, AK, BP, and SD interpreted results of experiments; HP and BP drafted manuscript; HP, MI, MG, AK, BP, and SD edited and revised manuscript and approved final version of manuscript.

## Conflict of Interest Statement

The authors declare that the research was conducted in the absence of any commercial or financial relationships that could be construed as a potential conflict of interest.
